# Possible *Exiguobacterium sibiricum* Skin Infection in Human

**DOI:** 10.3201/eid2012.140493

**Published:** 2014-12

**Authors:** Daniel Tena, Nora Mariela Martínez, Josefa Casanova, Juan Luis García, Elena Román, María José Medina, Juan Antonio Sáez-Nieto

**Affiliations:** Hospital Universitario de Guadalajara, Guadalajara, Spain (D. Tena, N.M. Martínez);; Centro de Salud de Molina de Aragón, Guadalajara (J. Casanova, J.L. García);; Servicios Periféricos de Salud y Bienestar Social, Guadalajara (E. Román);; Centro Nacional de Microbiología, Madrid, Spain (M.J. Medina, J.A. Sáez-Nieto)

**Keywords:** *Exiguobacterium* spp., *Exiguobacterium sibiricum*, cutaneous infection, cutaneous anthrax, bacteria

**To the Editor:** The genus *Exiguobacterium* was first described in 1983 by Collins et al., who characterized the species *E. aurantiacum* (*1*). Since then, 9 new species have been added: *E. acetylicum*, *E. antarcticum*, *E. undae*, *E. oxidotolerans*, *E. aestuarii*, *E. marinum*, *E. mexicanum*, *E. artemiae*, and *E. sibiricum* (*2*,*3*). The genus *Exiguobacterium* belongs to the group of coryneform bacteria, which encompasses aerobically growing, non–spore-forming, irregularly shaped, gram-positive rods (*2*). *Exiguobacterium* spp. have been isolated from a wide range of habitats, including cold and hot environments (*3*). Although strains of *Exiguobacterium* spp. have been isolated from human clinical specimens (e.g., skin, wounds, and cerebrospinal fluid), the clinical significance of these bacteria is poorly understood (*4*). We present a case of cutaneous infection possibly caused by *E. sibiricum*.

In January 2014, a previously healthy 66-year-old farmer was admitted to the Health Center of Molina de Aragón (Guadalajara, Spain) with a 7-day history of an ulcer on the dorsal surface of the second finger on his right hand with a painful black eschar surrounded by edema, greenish exudate, erythema, and a broken blister. The lesion had progressively increased in size. The patient was a hunter who had handled the skin of a deer and a wild boar 4 days before. He had no history of trauma or receipt of antimicrobial drugs. At admission, he was afebrile with no systemic symptoms. Cutaneous anthrax was suspected on the basis of the clinical appearance of the lesion and the patient’s contact with animals. An exudate sample was obtained for culture, and treatment with oral ciprofloxacin (500 mg/12 hour) was initiated. The Gram-stained sample showed leukocytes without organisms. Culture was performed according to standard practice. 

Colonies observed after 24 hours of incubation on blood agar in pure culture were gray but turned orange after 48 hours. The colonies appeared mucoid and were nonhemolytic. Gram staining revealed wide, short, non–spore-forming, gram-positive rods. The isolate was motile, catalase positive, oxidase negative, and it fermented glucose and lactose. Reactions for indole, urea, and bile esculin were negative. The strain did not grow on McConkey agar and was facultatively anaerobic. The strain was initially identified as *Bacillus* spp. and was sent to the National Reference Laboratory of Majadahonda (Madrid, Spain) for species identification. There, the isolate was identified as *E. sibiricum* by means of 16S rRNA sequence analysis according to a previously reported method (*5*). The fragment of 16S RNA gene obtained from this isolate was 1,413 bp, and similarity with GenBank sequences was 99.6% (GenBank accession nos. CP00122, GQ869573, and others). 

After the organism was identified, we found that it was able to grow on blood agar at 4°C after 6 days of incubation. Antimicrobial drug susceptibility testing was performed by using the Etest method (AB Biodisk, Solna, Sweden) on Mueller-Hinton agar plates incubated at 37°C for 24 hours. The isolate was susceptible to penicillin (MIC 0.023 mg/L), cefotaxime (0.5 mg/L), imipenem (0.047 mg/L), levofloxacin (0.19 mg/L), vancomycin (0.5 mg/L), clindamycin (0.125 mg/L), erythromycin (0.047 mg/L), gentamicin (0.094 mg/L), doxycycline (0.032 mg/L), linezolid (0.5 mg/L), and daptomycin (0.5 mg/L). The patient’s clinical outcome was good, and the lesion resolved after 10 days of continuous ciprofloxacin therapy.

This patient’s cutaneous infection and the morphologic appearance of the lesion resembled cutaneous anthrax. Initially, the Gram-stained appearance and culture were compatible with those of *Bacillus* species other than *B. anthracis*. In this sense, cutaneous infections caused by *Bacillus* species other than *B. anthracis* have been reported and are clinically similar to cutaneous anthrax (*6*). Isolation of coryneform bacteria from the ulcer may represent colonization rather than true infection, and the absence of the organism on the initial Gram-stained slides may support contamination. However, the evidence points to *E. sibiricum* as a pathogen and not a contaminant because it was the only organism isolated, and Gram staining of the exudate revealed leukocytes. In addition, the patient had not previously received any antimicrobial drug that could change the result of the culture. Moreover, the isolate was susceptible to ciprofloxacin, and clinical response to this drug was good. However, we cannot absolutely rule out another organism as the cause of the infection or co-infection with some uncultured bacterium.

Identification of *Exiguobacterium* spp. based on conventional methods is difficult and should be confirmed with molecular assays. Bacteria in this genus can be misidentified as *Oerskovia xanthineolytica* when the API Coryne kit (bioMérieux, Marcy l’Étoile, France) is used (*7*); 16S rRNA gene sequencing seems to be useful for identification of *E. sibiricum* (*8*). Consequently, the frequency of this infection can be underdiagnosed. In patients with lesions suspected of being cutaneous anthrax, *E. sibiricum* should be considered as a potential cause and should be differentiated from *B. anthracis* (Table) (*9*,*10*).

**Table:** Microbiological and clinical characteristics of *Exiguobacterium sibiricum* and Bacillus anthracis*

Characteristic	*E. sibiricum*	*B. anthracis*
Colony on blood agar	Mucoid and orange	Gray-white to white
Spore production	–	+ (central)
Motility	+	–
Hemolysis on blood agar	–	–
Penicillin susceptibility	+	+
Catalase production	+	+
Indole production	–	–
Growth at 4°C	+	–
Anaerobic growth	+	+
Cutaneous infection	Ulcer, black eschar, blister	Eschar, malignant pustule
Other infections	None reported	Intestinal anthrax, pulmonary anthrax, meningitis

*+, present; –, absent.

This case of human infection was most likely caused by *E. sibiricum*. Identification of this organism is difficult, and it can be confused with *Bacillus* spp. *E. sibiricum* should be considered as a possible cause of lesions suspected of being cutaneous anthrax.
